# The efficacy and safety of tislelizumab combined with gemcitabine plus cisplatin in the treatment of postoperative patients with muscle-invasive upper tract urothelial carcinoma

**DOI:** 10.1186/s12885-024-11919-1

**Published:** 2024-02-13

**Authors:** Jingwen Zhang, Meng Yang, Dongqun Wei, Deru Zhang, Zeyu Chen, Haitao Zhu

**Affiliations:** Department of Urology, The Affiliated Hospital of Xuzhou Medical University, Xuzhou, China

**Keywords:** Tislelizumab, Gemcitabine plus cisplatin, Upper tract urothelial carcinoma, Efficacy, Safety

## Abstract

**Background:**

A combination of immune checkpoint inhibitors (ICIs) and chemotherapy has demonstrated excellent clinical efficacy and safety in treating a variety of cancers, including urothelial carcinoma (UC). However, its efficacy and safety in patients with muscle-invasive upper tract urothelial carcinoma (UTUC) who are undergoing radical surgery remain uncertain. The purpose of this retrospective study was to examine the effectiveness and safety of tislelizumab combined with gemcitabine plus cisplatin (TGC) as a first-line postoperative adjuvant treatment in this population.

**Methods:**

This single-center, real-world study retrospectively analyzed the data from 71 patients with muscle-invasive UTUC who had radical nephroureterectomy (RNU) at the Affiliated Hospital of Xuzhou Medical University between November 1, 2020, and November 1, 2023. Among the 71 patients, 30 received adjuvant therapy of TGC within 90 days after RNU and 41 underwent surveillance. No patients receive preoperative neoadjuvant therapy. The TGC therapy group received adjuvant therapy every 3 weeks postoperatively until the first recurrence, first metastasis, or death due to any reason, whichever occurred first. The patients were followed up telephonically and through outpatient visits to record and evaluate their disease-free survival (DFS) and treatment-related adverse events (TRAEs).

**Results:**

This study assessed the DFS of 41 and 30 patients in the surveillance group and TGC therapy group, respectively. The median DFS of the surveillance group was 16.5 [95% confidence interval (CI), 14.7–18.3] months, while the median DFS of the TGC group has not yet reached [hazard ratio (HR) 0.367 (95% CI, 0.169–0.796); *p* = 0.008], with 21 patients still undergoing follow-up. Compared with the surveillance group, the TGC therapy group had dramatically improved DFS after RNU and reduced risk by 63.3%. Of the 30 patients receiving combination therapy, 28 experienced TRAEs; all TRAEs were consistent with the frequently reported events in the chemotherapy-alone regimens, and there were no treatment-related deaths.

**Conclusion:**

This study demonstrates that TGC therapy exhibits excellent clinical efficacy in patients undergoing radical surgery, significantly improving DFS and displaying great safety.

**Supplementary Information:**

The online version contains supplementary material available at 10.1186/s12885-024-11919-1.

## Background

Urothelial carcinoma (UC) ranks sixth among common tumors in developed countries, among which, upper tract urothelial carcinoma (UTUC) accounts for 5-10%, and its annual incidence rate is about 2 cases per 100 000 inhabitants [1]. Despite being a relatively rare disease, its global incidence and fatality rates continue to rise annually, according to estimates from the American Cancer Society in 2023, the incidence of cases in the kidneys/renal pelvis and ureters/other urinary organs was 81,800 and 4470, respectively, with mortality rates of 14,890 and 990 (note: there is no separate breakdown for renal pelvis and ureter only), both showing an increase compared to previous years [2]. UTUC is more vulnerable to infiltration and growth because of the disease’s hazy early symptoms and the thin muscular layer of the ureter and renal pelvis compared to that of the bladder. Approximately two-thirds of patients with UTUC already have muscle layer infiltration upon first diagnosis [1], and the postoperative survival rate of patients with muscle-invasive UTUC is reportedly poor [3, 4]. Presently, radical nephroureterectomy (RNU) is the gold standard for treating UTUC; however, surgical treatment alone cannot be relied upon for individuals with muscle involvement because some patients may still have lethal metastasis and recurrence even after radical surgery [1]. Adjuvant chemotherapy has shown great improvement in patients’ prognosis following RNU [5–7], and because of its good tolerability, gemcitabine plus cisplatin (GC) has emerged as the standard chemotherapy regimen for urothelial carcinoma [1]. However, since chemotherapy is susceptible to drug resistance [8, 9], some patients may experience disease progression shortly after surgery, posing a threat to their prognosis. Therefore, it is crucial to investigate new therapeutic options to assist patients in managing this relatively uncommon condition to significantly increase their survival rate.

Recently, the discovery of programmed death 1 (PD-1) and associated research have revealed that immunological escape plays a major role in the development of tumors. PD-1 and its ligand 1 (PD-L1) participate in immune escape of tumor cells. Immune checkpoint inhibitor-based immunotherapy that targets PD-1 and PD-L1 offers patients therapeutic alternatives and has been effectively used to treat various malignancies, including urothelial carcinoma [10–13]. Platinum-based chemotherapy can boost the concomitant blocking effects of PD-1 and PD-L1 while also inducing immune regulatory effects [14]. Therefore, researchers are considering whether immunotherapy can be combined with chemotherapy to enhance clinical efficacy, and combination therapy is gradually gaining popularity. Thus far, the application of combination therapy for various cancers and its related research results have shown that it has good clinical efficacy and safety [15–17]. Multiple studies have used different immune checkpoint inhibitors (ICIs) combined with platinum chemotherapy to treat urothelial carcinoma and have reported positive clinical outcomes [18, 19].

Tislelizumab is another extensively used PD-1 monoclonal antibody in the clinical treatment of a variety of malignancies and has demonstrated good clinical efficacy and safety [20]. It has a high affinity and binding specificity for PD-1 and can block the interaction between PD-1 and its ligand, terminate the PD-1 immunosuppressive signal caused by the interaction between PD-1 and PD-L1 in T cells, and restore the immune response against tumors, thereby enhancing the killing effect on tumor cells [21]. Tislelizumab has been shown to have a higher affinity for PD-1, a slower dissociation rate, and a longer duration of action compared to other monoclonal antibodies [22], thereby allowing it to have great clinical efficacy. Therefore, combination therapy with tislelizumab and GC chemotherapy may have broad clinical treatment prospects. A combination of tislelizumab and platinum-based chemotherapy achieves excellent clinical efficacy and tolerance in patients with various cancers, including advanced lung cancer [23], esophageal squamous cell carcinoma, gastric/gastroesophageal junction adenocarcinoma [24], and bladder cancer [25]. Nevertheless, research on the effectiveness and safety of tislelizumab combined with gemcitabine plus cisplatin (TGC) in patients undergoing radical surgery for UTUC has not yet been conducted. Therefore, we added tislelizumab to the gemcitabine plus cisplatin regimen and used TGC therapy as a first-line adjuvant treatment for postoperative muscle-invasive UTUC patients, filling the gap in the effectiveness of TGC therapy for postoperative UTUC patients. This single-center retrospective study aims to observe the effectiveness and safety of TGC therapy in the treatment of postoperative UTUC patients, providing a reference for clinical treatment plans.

## Methods

### Information gathering

By reviewing hospital medical records, clinical data from patients with muscle-invasive UTUC who received RNU at the Affiliated Hospital of Xuzhou Medical University between November 1, 2020, and November 1, 2023, were collected and analyzed. The inclusion criteria were as follows: (1) Eligible patients should be at least 18 years old; (2) preoperative computed tomography urography (CTU) examination and ureteroscopy showing UTUC, with absent metastasis on chest, abdominal and pelvic computed tomography (CT); (3) patient has undergone laparoscopic RNU, including resection of all radiologically or macroscopically abnormal nodes; (4) a confirmed postoperative pathological diagnosis of muscle-invasive UTUC (pT2-pT4, Nany or lymph node positive pTany, N1-3, without metastasis M0) and negative surgical margins (R0); (5) start treatment within 90 days after surgery, at least three cycles of full dose TGC therapy performed; and (6) an Eastern Cooperative Oncology Group (ECOG) physical fitness status of 0/1. The exclusion criteria were: (1) Patients who received other immune drugs or therapies simultaneously; (2) patients with concurrent malignant tumors; (3) preoperative neoadjuvant therapy; and (4) hematology examination shows estimated glomerular filtration rate (eGFR) < 45 ml/min or patients lost to follow-up. The tumor staging was determined based on the American Joint Committee on Cancer (AJCC) TNM classification. Ultimately, 71 eligible patients (30 and 41 received TGC therapy and surveillance, respectively) were included in the analysis. Patients in the TGC group received tislelizumab (200 mg intravenous injection on day 7 of each cycle), gemcitabine (1000 mg/m2 intravenous injection on day 1 and 8 of each cycle), and cisplatin (70 mg/m2 intravenous injection on day 2 of each cycle) within 90 days postoperatively for 3 weeks as a treatment cycle, and all patients received at least three cycles of combination therapy. The presence or absence of treatment-related adverse events (TRAEs) were recorded through consultation and telephonic follow-up and graded according to the National Cancer Institute Adverse Event Terminology Standard 4.03. Pre- and post-therapy, adjuvant measures, including hydration, acid suppression, and antiemesis, were administered, and any adverse effects were handled appropriately. Before starting combination therapy, all patients had normal hematological and physical examination results and provided written informed consent. All procedures adhered to the principles of the Helsinki Declaration, and the research protocol was approved by the Ethics Committee of the Affiliated Hospital of Xuzhou Medical University.

### Follow up criteria and study endpoints

All included patients were followed up per the guidelines of the European Association of Urology [1]. For the first 2 years of treatment, follow-up was done every 3 months; for the third year, it was done every 6 months. Regular physical examinations, hematological exams, and cystoscopies were carried out for assessment over the follow-up period. Annual chest, abdominal and pelvis CT scans were performed to evaluate tumor recurrence and distant metastases; on the doctor’s recommendation, positron emission tomography/computed tomography (PET/CT) examinations were also performed for some patients. Disease-free survival (DFS), defined as the time from surgery to the occurrence of first recurrence, first metastasis, or death from any cause (tumor recurrence or metastasis is evaluated using the Solid Tumor Efficacy Evaluation Criteria 1.1 [26]), whichever occurs first, was the primary endpoint of this study. The secondary endpoint was TRAEs.

### Control for bias or confounding factors

To ensure the authenticity and reliability of the research results, we have taken various measures to reduce bias and confounding factors. Firstly, strict inclusion and exclusion criteria were established in this study to ensure that all patients included in the study met the requirements. When collecting patient information, we cross-checked to ensure the accuracy of the data. Secondly, we matched the two groups of patients included in the study and found no statistically significant difference in baseline characteristics between the two groups. In the end, we did not include too many confounding factors in multivariate Cox regression analysis, which reduced bias in the study and improved the accuracy of the research results.

### Statistical analysis

Fisher’s exact test or the chi-square test was used to compare the baseline characteristics of the two patient groups. TRAEs were summarized using descriptive statistics. The Kaplan–Meier (K-M) curves were used to estimate survival. Log-rank test was employed to assess statistically significant differences in DFS. Univariate Cox regression analysis was performed for all variables, and those with a *P* value of < 0.05 and potential confounding variables were then included in multivariate Cox regression analysis to examine the factors associated with DFS. Hazard ratio (HR) and 95% confidence interval (CI) were also calculated. Finally, we selected the following factors as variables in the multivariate Cox regression analysis: age, pathological T stage, lymph node metastasis, comorbidity number, and postoperative management. SPSS version 26.0 was used for all statistical analyses, and the difference was deemed statistically significant when the bilateral *P* value was less than 0.05.

## Results

### Patient demographics

**Fig. 1:**
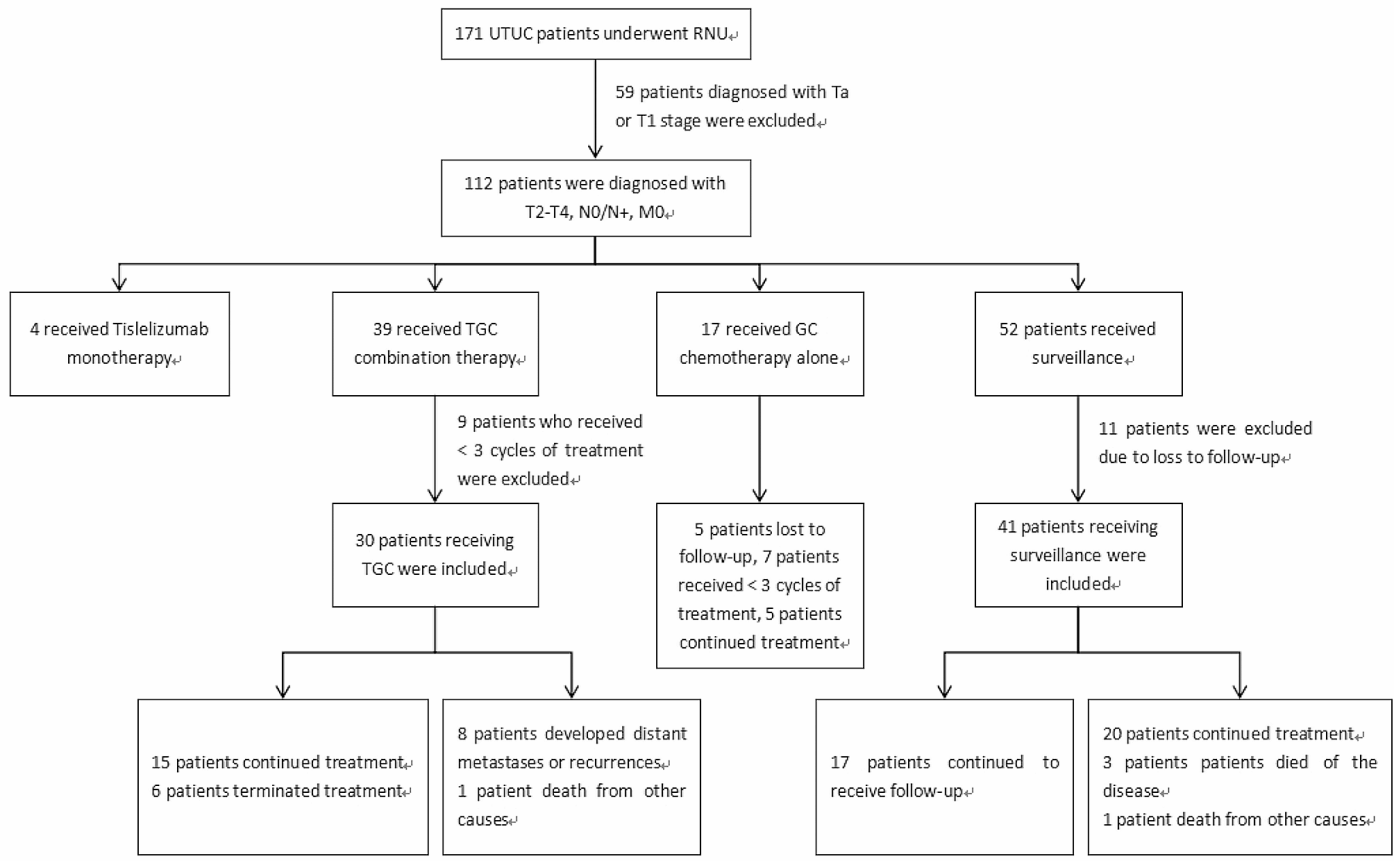
Patient screening process UTUC: upper tract urothelial carcinoma RNU: radical nephroureterectomy TGC: tislelizumab combined with gemcitabine plus cisplatin GC: gemcitabine plus cisplatin

A total of 171 patients with UTUC underwent RNU between November 2020 and November 2023. Among them, 59 patients with postoperative pathological assessment of Ta or T1 stage were not included. Among the remaining 112 patients, 39 received TGC combination therapy, 4 received tislelizumab monotherapy, 17 received GC chemotherapy alone, and 52 patients received surveillance; 9 patients in the TGC group had a combined medication cycle of less than three and 11 patients lost to follow-up in the surveillance group were excluded from the analysis. Finally, a total of 30 and 41 patients who received combined treatment and surveillance, respectively, were included in this study. As of November 2023, 15 patients in the TGC group continued treatment, 6 terminated treatment, and 9 terminated follow-up due to distant metastasis or recurrence (*n* = 8) or death from other causes (*n* = 1); 17 patients in the surveillance group continued to receive follow-up, while 24 patients terminated follow-up due to distant metastasis or recurrence (*n* = 20), death caused by this disease (*n* = 3), and death due to other reasons (*n* = 1) (Fig. 1). The median age of all patients included in the study was 75.0 years [InterQuartile Range (IQR) 66.0–78.0]. Table 1 shows the baseline characteristics of both groups; there were no significant differences in age, gender, ECOG status, smoking status, hematuria status, tumor size, tumor grade, pathological T stage, lymph node metastasis, tumor location, number of comorbidities, and eGFR between the two groups. It should be noted that although 11 patients in the TGC group had eGFR < 60 ml/min, their hematological examination before each combination therapy showed eGFR > 50 ml/min, and previous studies have shown that eGFR > 50 ml/min meets the criteria for using cisplatin [1, 6]. Therefore, they received full-dose cisplatin treatment.

**Table 1:** The baseline characteristics of the two groups of patients

	Total n = 71(100%)	TGC n = 30(42.3%)	Surveillance n = 41(57.7%)	χ″	*P*
Age,years,n(%)				1.295	0.255
≤ 75	37(52.1%)	18(60.0%)	19(46.3%)		
> 75	34(47.9%)	12(40.0%)	22(53.7%)		
Gender,n(%)				0.19	0.663
Male	47(66.2%)	19(63.3%)	28(68.3%)		
Female	24(33.8%)	11(36.7%)	13(31.7%)		
ECOG status,n(%)				0.024	0.878
0	49(69.0%)	21(70.0%)	28(68.3%)		
1	22(31.0%)	9(30.0%)	13(31.7%)		
Tobacca use,n(%)				0.415	0.52
Current or former	41(57.7%)	16(53.3%)	25(61.0%)		
Never	30(42.3%)	14(46.7%)	16(39.0%)		
Hematuria,n(%)				0.019	0.89
Yes	55(77.5%)	23(76.7%)	32(78.0%)		
No	16(22.5%)	7(23.3%)	9(22.0%)		
Tumor size,n(%)				2.011	0.156
≤ 3	33(46.5%)	11(36.7%)	22(53.7%)		
> 3	38(53.5%)	19(63.3%)	19(46.3%)		
Grade,n(%)				0.102	0.617
High	68(95.8%)	29(96.7%)	39(95.1%)		
Low	3(4.2%)	1(3.3%)	2(4.9%)		
Pathological stage,n(%)				0.256	0.914
T2	24(33.8%)	11(36.7%)	13(31.7%)		
T3	44(62.0%)	18(60.0%)	26(63.4%)		
T4	3(4.2%)	1(3.3%)	2(4.9%)		
Lymph node metastasis,n(%)				0.706	0.401
pN0	64(90.1%)	26(86.7%)	38(92.7%)		
pN+	7(9.9%)	4(13.3%)	3(7.3%)		
Location,n(%)				0.665	0.415
Renal pelvis	30(42.3%)	11(36.7%)	19(46.3%)		
Ureter	41(57.7%)	19(63.3%)	22(53.7%)		
Comorbidity				2.545	0.111
number,n(%)	47(66.2%)	23(76.7%)	24(58.5%)		
≤ 2	24(33.8%)	7(23.3%)	17(41.5%)		
> 2					
eGFR,ml/min,n(%)				1.033	0.309
< 60	31(43.7%)	11(36.7%)	20(48.8%)		
≥ 60	40(56.3%)	19(63.3%)	21(51.2%)		

TGC: tislelizumab combined with gemcitabine plus cisplatin

ECOG: eastern cooperative oncology group

pN0: no lymph node involvement

pN+: lymph node involvement

eGFR: estimated glomerular filtration rate

### Clinical efficacy and safety of TGC combination therapy

**Fig. 2:**
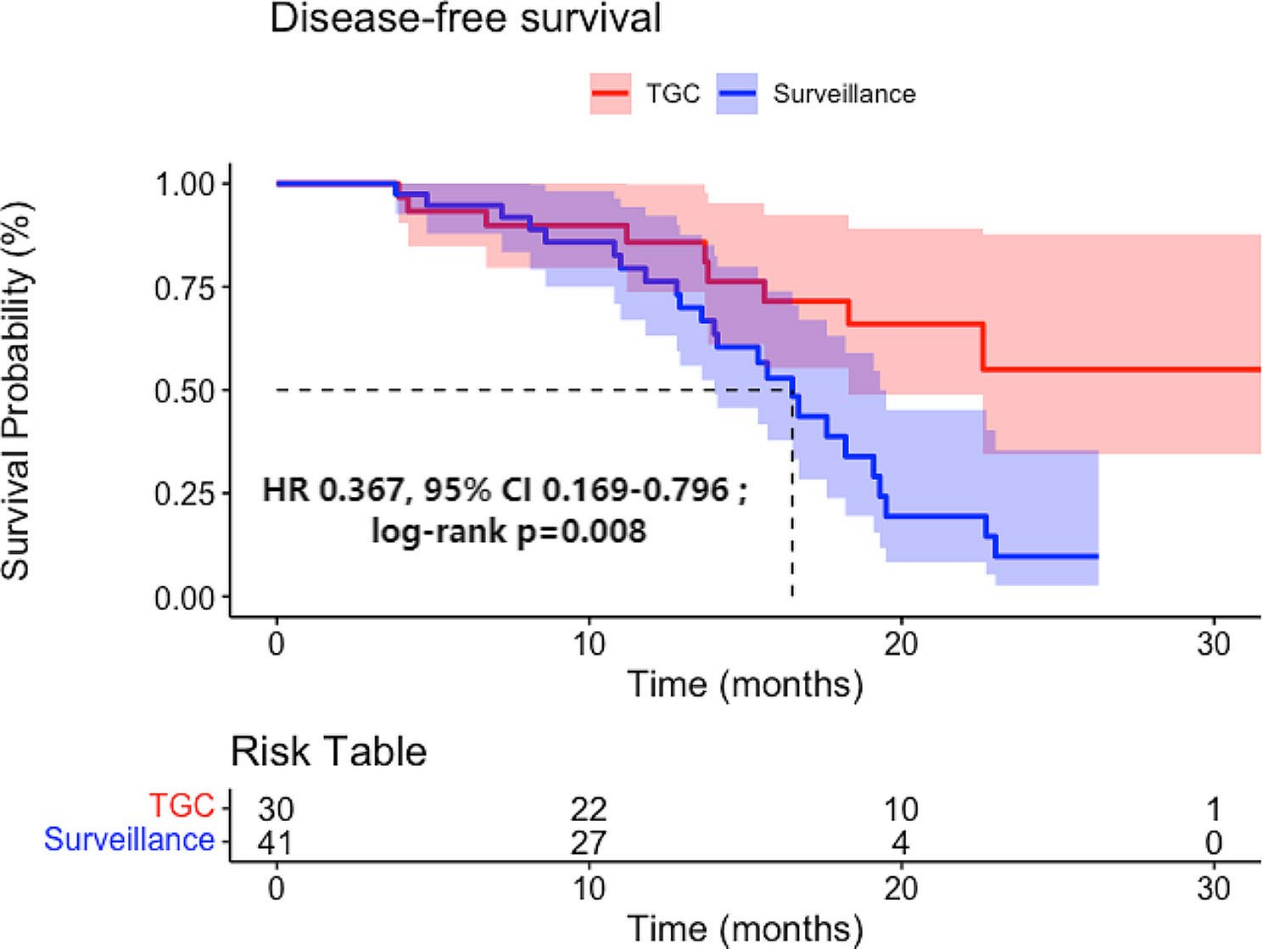
Comparison of disease-free survival between patients in the tislelizumab combined with gemcitabine plus cisplatin chemotherapy (TGC) group and the Surveillance group. The median DFS of the surveillance group was 16.5 [95% confidence interval (CI), 14.7–18.3] months, while the median DFS of the TGC group has not yet been reached [hazard ratio (HR) 0.367 (95% CI, 0.169–0.796); *p* = 0.008]. Compared with the surveillance group, the combination therapy group had dramatically improved DFS after RNU and reduced risk by 63.3% TGC: tislelizumab combined with gemcitabine plus cisplatin HR: hazard ratio CI: confidence interval

**Fig. 3:**
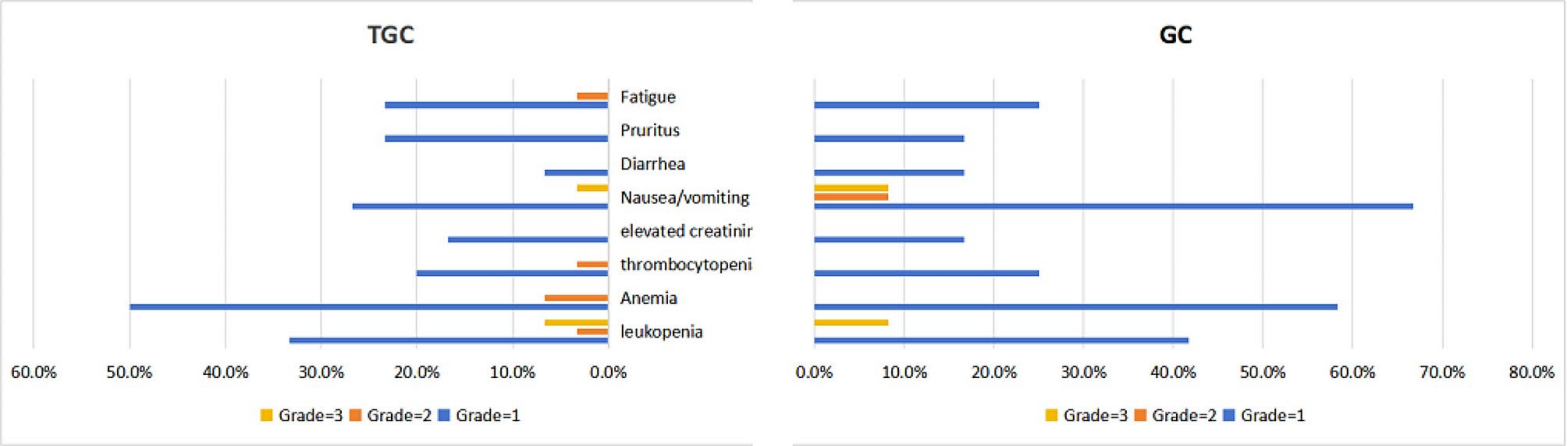
Comparison of TRAEs between TGC group and GC group TGC: tislelizumab combined with gemcitabine plus cisplatin GC: gemcitabine plus cisplatin

As of November 1, 2023, patients receiving combination therapy (9 [30.0%] of 30) reported fewer primary endpoint events due to the disease compared to those receiving surveillance (24 [58.5%] of 41). The median DFS (mDFS) follow-up times for the TGC and surveillance groups were 15.8 (IQR 9.6–21.0) months and 14.00 (IQR 7.65–16.60) months, respectively. The mDFS for the surveillance group was 16.5 (95% CI 14.7–18.3) months. Although the mDFS follow-up time for this study was relatively long, the mDFS for the TGC group has not yet been reached. In addition, compared to the surveillance group, the absolute risk reduction (ARR) rate of the TGC group was 28.5%, demonstrating excellent clinical efficacy. Univariate Cox regression analysis was performed for all variables, and those with a *P* value of < 0.05 and potential confounding variables were then included in multivariate Cox regression analysis to examine the clinical characteristics associated with DFS (Table 2). Multivariate Cox regression analysis showed a significant correlation between postoperative combination therapy and the benefits of DFS. The K-M survival curve was used to analyze the differences in patient survival outcomes (Fig. 2), and the results showed that compared to the surveillance group, the TGC group reduced the relative risk of disease recurrence, metastasis, or death by 63.3% [HR 0.367 (95% CI 0.169–0.796); *p* = 0.008], significantly prolonging the DFS of patients and improving their prognosis. The total incidence of TRAEs in the combination therapy group was 93.3% (28/30), including 13 cases of leukopenia, 17 cases of anemia, 7 cases of thrombocytopenia, 5 cases of elevated creatinine (all occur in patients with eGFR < 60 ml/min), 9 cases of nausea or vomiting, 2 cases of diarrhea, 7 cases of pruritus, and 8 cases of fatigue. Among these, there were 21, 4, and 3 cases of grade 1, 2, and 3 adverse events (manifested as total parenteral nutrition and a white blood cell count of < 2.0 × 10^9^/L), respectively; and there were no cases of immune myocarditis or severe adverse events, such as pancreatitis and TRAEs related deaths (Table 3). The most common TRAEs were anemia, leukopenia, and nausea or vomiting, which are consistent with the adverse events related to combination therapy reported in previous literature. The total incidence of TRAEs in the GC group was 100% (12/12), including 5 cases of leukopenia, 7 of anemia, 3 of thrombocytopenia, 2 of elevated creatinine, 8 of nausea and vomiting, 2 of diarrhea, 2 of itching, and 3 of fatigue. Among these, there were 9, 1, and 2 cases of grade 1, 2, and 3 TRAEs (manifested as total parenteral nutrition and white blood cell count < 2.0 × 109/L), respectively; no deaths related to TRAEs were reported (Table 4). Figure 3 shows the comparison of TRAEs between TGC and GC group.

**Table 2:** Univariate and Multivariable Cox model for disease-free survival

Variables	Disease-free survival			
	Univariate analyses Hazard Ratios (95% CI)	*P*	Multivariate analyses Hazard Ratios (95% CI)	*P*
Age	1.927(0.938–3.961)	0.074	1.271(0.555–2.910)	0.571
Gender	0.985(0.488–1.990)	0.967		
ECOG status	1.549(0.746–3.128)	0.24		
Tobacca use	1.152(0.578–2.293)	0.688		
Hematuria	0.703(0.334–1.478)	0.353		
Tumor size	1.041(0.523–2.072)	0.908		
Grade	1.257(0.299–5.282)	0.754		
Pathological stage				
T2	1.000(Ref.)		1.000(Ref.)	
T3	3.056(1.299–7.191)	0.01	2.968(1.197–7.357)	0.019
T4	50.561(10.038–254.680)	< 0.001	26.557(3.497-201.692)	0.002
Lymph node metastasis	3.293(1.322–8.208)	0.011	1.405(0.407–4.859)	0.591
Location	0.676(0.340–1.345)	0.265		
Comorbidity number	2.565(1.276–5.154)	0.008	2.316(1.069–5.017)	0.033
eGFR	1.413(0.700-2.855)	0.335		
Postoperative management	0.367(0.169–0.796)	0.011	0.387(0.164–0.911)	0.03

CI: confidence interval

ECOG: eastern cooperative oncology group

eGFR: estimated glomerular filtration rate

**Table 3:** The incidence of TRAEs in TGC group

	TGC(*n* = 30)		
	Grade = 1	Grade = 2	Grade = 3
Patients with TRAEs	21(70.0%)	4(13.3%)	3(10%)
leukopenia	10(33.3%)	1(3.3%)	2(6.7%)
Anemia	15(50.0%)	2(6.7%)	0
thrombocytopenia	6(20.0%)	1(3.3%)	0
elevated creatinine	5(16.7%)	0	0
Nausea/vomiting	8(26.7%)	0	1(3.3%)
Diarrhea	2(6.7%)	0	0
Pruritus	7(23.3%)	0	0
Fatigue	7(23.3%)	1(3.3%)	0

TGC: tislelizumab combined with gemcitabine plus cisplatin

TRAEs: treatment related adverse events

**Table 4:** The incidence of TRAEs in GC group

	GC(*n* = 12)		
	Grade = 1	Grade = 2	Grade = 3
Patients with TRAEs	9(75.0%)	1(8.3%)	2(16.7%)
leukopenia	5(41.7%)	0	1(8.3%)
Anemia	7(58.3%)	0	0
thrombocytopenia	3(25.0%)	0	0
elevated creatinine	2(16.7%)	0	0
Nausea/vomiting	8(66.7%)	1(8.3%)	1(8.3%)
Diarrhea	2(16.7%)	0	0
Pruritus	2(16.7%)	0	0
Fatigue	3(25.0%)	0	0

GC: gemcitabine plus cisplatin

TRAEs: treatment related adverse events

### Limitations of research findings

As a novel immunotherapy drug, tislelizumab has not yet been widely used in clinical treatment. Therefore, the sample size included in this study is small, which may lead to a lack of representativeness for postoperative UTUC patients, reducing the general applicability and accuracy of the study. A small sample size may also lead to confounding factors having a greater impact on the results, limiting the application of multivariate analysis. Therefore, it is necessary to increase the sample size in order to improve the accuracy of research results and the reliability of clinical applications. In addition, as a retrospective study, incomplete clinical data or many potential biases and confounding factors may affect the final results.

## Discussion

Although the incidence of UTUC is low, the probability of muscle infiltration is high. For patients who present with muscle layer infiltration at initial diagnosis, the prognosis is poor [3, 4]. Currently, the majority of research indicates that postoperative adjuvant chemotherapy can enhance the possibility of survival of patients with UTUC. A study involving 126 patients with UTUC with stage pT2-4 N0/X M0 showed that adjuvant chemotherapy can improve the prognosis of advanced UTUC at a median follow-up interval of 23.6 months [5]. Another phase 3 clinical trial called POUT included 261 patients with stage pT2-4 N0-3 M0 or pTany N1-3 M0 UTUC and demonstrated that adjuvant chemotherapy considerably enhanced DFS and lowered the relative risk of disease recurrence or death by 55% at a median follow-up 30.3 months [6]. Recently, ICIs have shown good therapeutic effects clinically. In a phase 3 clinical trial, patients who underwent radical cystectomy or RNU and received nivolumab had a nearly two-fold longer DFS than those who received a placebo at a median follow-up of 20.9 months [10]. Based on the excellent therapeutic effects demonstrated by chemotherapy and ICIs, a combination of ICIs and GC is being used clinically for various cancers, including urothelial carcinoma. The use of TGC for the treatment of locally advanced or metastatic bladder cancer showed that when the median follow-up period was 54.3 weeks, patients in the TGC group had a better prognosis than those in the GC group [25].

The present study retrospectively investigated the clinical efficacy and safety of TGC as a first-line adjuvant treatment for patients with UTUC undergoing radical surgery. As of November 2023, the patients receiving combined therapy had a longer mDFS than those who received only surveillance. Although the mDFS of the TGC group in this study has not yet been achieved, the risk decreased by 63.3% compared to the surveillance group [HR 0.367 (95% CI 0.169–0.796); *p* = 0.008], which is better than the results of adjuvant chemotherapy alone in the POUT experiment. This could be because some patients in the POUT experiment received gemcitabine plus carboplatin as the chemotherapy regimen; a study suggests that compared to gemcitabine plus carboplatin, a combination of gemcitabine and cisplatin may have better clinical efficacy [27]. It is also possible that platinum-based chemotherapy induces immune regulatory effects while enhancing the accompanying PD-1 and PD-L1 blocking effects [14], generating synergistic effects and increasing clinical efficacy. The GC regimen, as a first-line adjuvant treatment for upper tract urothelial carcinoma after radical surgery, has achieved good clinical efficacy. Based on this, this study added tislelizumab and used the TGC regimen as a first-line adjuvant treatment for postoperative UTUC patients, achieving better therapeutic effects. This further improves the clinical prognosis of patients, provides a new treatment strategy for patients, and also provides a more excellent treatment choice for clinical doctors.

In the present study, the T-stage of tumors and the number of comorbidities the patient had were also independent risk factors affecting the prognosis of DFS, which is consistent with the findings of previous studies. A multicenter, retrospective study involving 1363 patients receiving RNU treatment showed a significant correlation between pathological T staging and postoperative survival at a median follow-up time of 37.2 months [28]. Despite our small sample size, our results confirm the conclusions of the aforementioned study. However, in the present study, the CI corresponding to the T4 phase in the multivariate Cox regression analysis was relatively large, which the researchers believe was due to the statistical bias caused by the small sample size of the T4 phase patients. Moreover, as the number of comorbidities in the patient increases, the decline and weakness of their physical fitness leads to a corresponding decrease in their tolerance to TGC combination therapy, ultimately resulting in a poorer prognosis. An increase in the number of comorbidities has been reported to decrease the likelihood that the patient will receive adjuvant chemotherapy [29]. In our study, the patients in the dynamic observation group may have difficulty tolerating postoperative adjuvant chemotherapy due to their increased comorbidities and physical weakness. Another study involving 428 patients with UTUC who received adjuvant chemotherapy within 90 days after RNU surgery and had a pathological stage of pT2-4 N0/X M0 showed that the presence of few comorbidities significantly improved the overall survival and cancer-specific survival [30].

No new TRAEs were observed in the TGC group, and they were all within the tolerable range. Thyroid dysfunction is a common adverse event associated with immunotherapy [20, 31]; however, no thyroid dysfunction was observed in the patients in this study. This may be due to the small sample size or the delayed occurrence of adverse events, which were not observed during the follow-up period. Notably, in the present study, there were no deaths linked to TRAEs or significant adverse events associated with treatment. The TRAEs that occurred during treatment were tolerable and controllable and did not affect the patient’s quality of life in the short term. Furthermore, due to the short follow-up time of this study, only short-term TRAEs can be observed, and late-onset adverse events and their corresponding management measures cannot be predicted. This may lead to a lack of monitoring for adverse events, affecting the research results. In the future, studies should be conducted with longer follow-up times to verify the long-term safety of combination therapy.

The expression status of PD-L1, a potential biomarker for identifying TGC treatment response, can predict the prognosis of patients to a certain extent. A previous study has shown that patients with PD-L1 expression level of ≥ 1% have better prognosis [10]. Another study also showed that the PD-L1 expression level in patients has no significant impact on the postoperative application of immunotherapy [32]. Due to economic and other reasons, only 2 of the 30 patients (6.7%) in this study underwent postoperative pathological PD-L1 expression evaluation. Therefore, one limitation of this study is that we did not include the postoperative pathological PD-L1 expression status of patients, which may lead to potential patients with higher PD-L1 expression levels in this study achieving better clinical efficacy after receiving combination therapy, affecting the final results. Markers such as interleukin-6, interleukin-8, and interferon-γ have also been confirmed to be able to predict the prognosis of patients to a certain extent [33]. Further research should focus on incorporating potential biomarkers in tumor tissue and explore their roles. The small sample size is another limitation of this study, which may lead to a lack of universal applicability and accuracy in the research results. A larger sample size study is needed to further verify the accuracy of the results. Moreover, all patients in this study did not undergo regional lymph node dissection intraoperatively, and only enlarged lymph nodes or locally metastatic lymph nodes that were detected on preoperative imaging were treated. Research indicates that even enlarged lymph node dissection following surgery does not improve the prognosis of patients with UTUC, rather, it raises the risk of complications, such as abdominal organ injury and vascular problems [34]. Some researchers also believe that retroperitoneal lymph node dissection is beneficial for the clinical prognosis of patients with UTUC [35]. In this study, the results of the multivariate Cox regression analysis showed that lymph node positivity was not an independent risk factor affecting DFS in patients. However, due to the limited sample size and inevitable selection bias, the current research results require additional validation. Finally, the total treatment cycle of patients in this study is not completely consistent, and it is unclear whether increasing the treatment cycle will further improve the prognosis of patients. This needs to be further validated in subsequent studies to guide the specific medication plan for combination therapy in clinical applications.

After further review and verification of hospital medical records and follow-up of patients, we found that among the 17 patients with UTUC who received separate GC chemotherapy after RNU surgery in this study, 5 were lost to follow-up, 7 received < 3 cycles of chemotherapy, and as of the deadline of this study, 5 patients were still receiving treatment. Finally, only 5 patients could be included in the group receiving GC chemotherapy alone. The researchers believe that a small sample size can cause selection bias, lack of representativeness for the overall population, lead to a decrease in the accuracy of the research results, and also reduce the replicability of the study. Therefore, the group receiving GC chemotherapy alone could not be included in this study. However, researchers conducted follow-up surveys on TRAEs in patients receiving GC chemotherapy and presented the data as charts, which to some extent confirm the similarity of TRAEs between the TGC combined therapy and GC treatment groups.

To the best of our knowledge, this is the first retrospective study to use TGC as the first-line adjuvant therapy for patients with UTUC who are undergoing RNU surgery. To some extent, this proves that TGC combined therapy has good clinical efficacy and safety, providing patients with a novel and effective treatment plan. This is also a successful practice of ICIs combined with platinum chemotherapy in clinical treatment. In the future, more research on combination therapy should be conducted to support its clinical feasibility.

TGC therapy has been proven to have excellent clinical efficacy in metastatic urothelial carcinoma [20] and has shown gratifying clinical efficacy as a neoadjuvant therapy scheme for muscle-invasive bladder cancer [36], helping patients improve their prognosis. Although this study confirms that TGC combination therapy can improve the prognosis of postoperative UTUC patients, its use as a neoadjuvant therapy for patients with muscle-invasive UTUC is yet to be evaluated. Bladder cancer and UTUC have numerous similarities in their clinical features and treatment plans; the success of TGC combined treatment in the direction of bladder cancer indicates, to some extent, its potential effectiveness as a neoadjuvant treatment option for patients with muscle-invasive UTUC. New clinical research needs to be carried out in the future to confirm the clinical efficacy and safety of TGC as a neoadjuvant treatment plan for patients with UTUC. Based on the limitations of this study, it is necessary to further extend the study duration or conduct multicenter studies to increase sample size and verify the long-term safety of combination therapy. In addition, potential biomarkers and treatment cycles of patients should be included in the study, and the relationship between these factors and clinical efficacy should be clarified to improve the accuracy of research results and the reliability of clinical applications, further guiding clinical medication plans.

## Conclusion

This retrospective study indicates that TGC combination therapy demonstrated good clinical efficacy in patients with muscle-invasive UTUC receiving RNU by significantly prolonging their DFS and generally being well tolerated. However, this experiment is a single-center, retrospective study with a small sample size and limited follow-up time, which may lead to some bias in the results. Further multicenter studies with larger sample sizes are needed to observe the clinical efficacy and safety of TGC therapy and provide reference for clinical treatment plans.

### Electronic supplementary material

Below is the link to the electronic supplementary material.

**Supplementary Material 1:** STROBE Statement—checklist of items that should be included in reports of observational studies

## Data Availability

No datasets were generated or analysed during the current study.

## Abbreviations

AJCC: American Joint Committee on Cancer
ARR: Absolute risk reduction
CI: Confidence interval
CT: Computed tomography
CTU: Computed tomography urography
DFS: Disease-free survival
ECOG: Eastern Cooperative Oncology Group
eGFR: Estimated glomerular filtration rate
GC: Gemcitabine plus cisplatin
HR: Hazard ratio
ICIs: Immune checkpoint inhibitors
IQR: InterQuartile Range
K- M: L- Kaplan–Meier
mDFS: Median disease-free survival
PD-1: Programmed death 1
PD-L1: Programmed death-ligand 1
PET/CT: Positron emission tomography/computed tomography
pN+: Lymph node involvement
pN0: No lymph node involvement
RNU: Radical nephroureterectomy
TGC: Tislelizumab combined with gemcitabine plus cisplatin
TRAEs: Treatment related adverse events
UC: Urothelial carcinoma
UTUC: Upper tract urothelial carcinoma
