# Supplementary material for: The efficacy and safety of tislelizumab combined with gemcitabine plus cisplatin in the treatment of postoperative patients with muscle-invasive upper tract urothelial carcinoma
Source: BMC Cancer. 2024 Feb 13;24:202. doi: 10.1186/s12885-024-11919-1 (PMC10863243; doi:10.1186/s12885-024-11919-1)
Supplement: Supplementary file 1 — Supplementary Material 1: STROBE Statement—checklist of items that should be included in reports of observational studies [file 12885_2024_11919_MOESM1_ESM.docx]

STROBE Statement—checklist of items that should be included in reports of observational studies

|  | Item No. | Recommendation | Page  No. | Relevant text from manuscript |
| --- | --- | --- | --- | --- |
| **Title and abstract** | 1 | (*a*) Indicate the study’s design with a commonly used term in the title or the abstract | 2 | retrospective study |
|  |  | (*b*) Provide in the abstract an informative and balanced summary of what was done and what was found | 3 | the combination of Tislelizumab and GC chemotherapy exhibits excellent clinical efficacy and safety |
| Introduction | | | |  |
| Background/rationale | 2 | Explain the scientific background and rationale for the investigation being reported | 3 | The prognosis for patients with UTUC is poor |
| Objectives | 3 | State specific objectives, including any prespecified hypotheses | 3 | TGC therapy can help patients with UTUC achieve a better prognosis |
| Methods | | | |  |
| Study design | 4 | Present key elements of study design early in the paper | 2 | Postoperative survival of all patients receiving radical nephroureterectomy |
| Setting | 5 | Describe the setting, locations, and relevant dates, including periods of recruitment, exposure, follow-up, and data collection | 6 | By reviewing hospital medical records and medical records, clinical data of patients with muscle-invasive UTUC who received RNU at the Affiliated Hospital of Xuzhou Medical University from November 1, 2020 to November 1, 2023 |
| Participants | 6 | (*a*) *Cohort study*—Give the eligibility criteria, and the sources and methods of selection of participants. Describe methods of follow-up  *Case-control study*—Give the eligibility criteria, and the sources and methods of case ascertainment and control selection. Give the rationale for the choice of cases and controls  *Cross-sectional study*—Give the eligibility criteria, and the sources and methods of selection of participants | N/A |  |
|  |  | (*b*) *Cohort study*—For matched studies, give matching criteria and number of exposed and unexposed  *Case-control study*—For matched studies, give matching criteria and the number of controls per case | 2 | Among all 71 patients, 30 patients received adjuvant therapy of TGC within 90 days after RNU; 41 patients underwent surveillance. |
| Variables | 7 | Clearly define all outcomes, exposures, predictors, potential confounders, and effect modifiers. Give diagnostic criteria, if applicable | 7.8 | DFS is defined as the time from surgery to the occurrence of first recurrence, first metastasis, or death from any cause |
| Data sources/ measurement | 8* | For each variable of interest, give sources of data and details of methods of assessment (measurement). Describe comparability of assessment methods if there is more than one group | 8 | Utilize the Fisher's exact test or the chi square test to compare the baseline characteristics of two patient groups. Summarize patient TRAEs using descriptive statistics. Apply the Kaplan-Meier (K-M) technique to the survival curve estimation. Log-rank test is employed to assess statistically significant differences in DFS. Multivariate Cox proportional hazards regression analysis is used to evaluate variables that affect patient prognosis. The 95% confidence interval and hazard ratio were also calculated. SPSS version 26.0 was used for all statistical analyses, and the difference is deemed statistically significant when the bilateral P value is less than 0.05. |
| Bias | 9 | Describe any efforts to address potential sources of bias | 6 | Apply strict criteria for patient inclusion |
| Study size | 10 | Explain how the study size was arrived at | 6 | The number of patients who meet the requirements of the study was screened retrospectively |

Continued on next page

| Quantitative variables | 11 | Explain how quantitative variables were handled in the analyses. If applicable, describe which groupings were chosen and why | 9 | Quantitative variables are grouped and transformed into qualitative variables |
| --- | --- | --- | --- | --- |
| Statistical methods | 12 | (*a*) Describe all statistical methods, including those used to control for confounding | 8 | Utilize the Fisher's exact test or the chi square test to compare the baseline characteristics of two patient groups. Summarize patient TRAEs using descriptive statistics. Apply the Kaplan-Meier (K-M) technique to the survival curve estimation. Log-rank test is employed to assess statistically significant differences in DFS. Multivariate Cox proportional hazards regression analysis is used to evaluate variables that affect patient prognosis. The 95% confidence interval and hazard ratio were also calculated. SPSS version 26.0 was used for all statistical analyses, and the difference is deemed statistically significant when the bilateral P value is less than 0.05 |
|  |  | (*b*) Describe any methods used to examine subgroups and interactions | N/A |  |
|  |  | (*c*) Explain how missing data were addressed | N/A |  |
|  |  | (*d*) *Cohort study*—If applicable, explain how loss to follow-up was addressed  *Case-control study*—If applicable, explain how matching of cases and controls was addressed  *Cross-sectional study*—If applicable, describe analytical methods taking account of sampling strategy | 2 | Patient-related data were collected retrospectively and grouped into different treatments |
|  |  | (*e*) Describe any sensitivity analyses | N/A |  |
| Results | | | | |
| Participants | 13* | (a) Report numbers of individuals at each stage of study—eg numbers potentially eligible, examined for eligibility, confirmed eligible, included in the study, completing follow-up, and analysed | 8.9 | A total of 171 UTUC patients underwent RNU between November 2020 and November 2023. Among them... |
|  |  | (b) Give reasons for non-participation at each stage | 8.9 | Flowcharts are used to illustrate the reasons for the exclusion of patients at each stage |
|  |  | (c) Consider use of a flow diagram | 10 | Figure 1 |
| Descriptive data | 14* | (a) Give characteristics of study participants (eg demographic, clinical, social) and information on exposures and potential confounders | 9 | Table 1 displays the baseline characteristics of the two groups of patients |
|  |  | (b) Indicate number of participants with missing data for each variable of interest | N/A |  |
|  |  | (c) *Cohort study*—Summarise follow-up time (eg, average and total amount) | N/A |  |
| Outcome data | 15* | *Cohort study*—Report numbers of outcome events or summary measures over time | *N/A* |  |
|  |  | *Case-control study—*Report numbers in each exposure category, or summary measures of exposure | 9 | Finally, a total of 30 patients receiving combined treatment and 41 patients receiving surveillance were included in this study |
|  |  | *Cross-sectional study—*Report numbers of outcome events or summary measures | *N/A* |  |
| Main results | 16 | (*a*) Give unadjusted estimates and, if applicable, confounder-adjusted estimates and their precision (eg, 95% confidence interval). Make clear which confounders were adjusted for and why they were included | 12 | Univariate Cox regression analysis was done on all variables, and variables with P values less than 0.05 were then chosen for multivariate Cox regression analysis in order to examine the clinical characteristics associated to DFS (Table 2) |
|  |  | (*b*) Report category boundaries when continuous variables were categorized | N/A |  |
|  |  | (*c*) If relevant, consider translating estimates of relative risk into absolute risk for a meaningful time period | N/A |  |

Continued on next page

| Other analyses | 17 | Report other analyses done—eg analyses of subgroups and interactions, and sensitivity analyses | N/A |  |
| --- | --- | --- | --- | --- |
| Discussion | | | | |
| Key results | 18 | Summarise key results with reference to study objectives | 12 | Multivariate Cox regression analysis showed a significant correlation between postoperative combination therapy and the benefits of DFS |
| Limitations | 19 | Discuss limitations of the study, taking into account sources of potential bias or imprecision. Discuss both direction and magnitude of any potential bias | 19 | Due to the limited number of patients with known PD-L1 expression status in this study... |
| Interpretation | 20 | Give a cautious overall interpretation of results considering objectives, limitations, multiplicity of analyses, results from similar studies, and other relevant evidence | 19.20 | Although the sample size of this study is small and the follow-up time is limited, it is not possible to include all factors in the multivariate model, the results of using Tislelizumab alone to treat urothelial carcinoma or the combination of Tislelizumab and platinum based chemotherapy to treat other tumors can confirm our research findings, supporting the feasibility and effectiveness of combination therapy for UTUC patients undergoing radical surgery |
| Generalisability | 21 | Discuss the generalisability (external validity) of the study results | 20 | this experiment is a single center retrospective study with a small sample size and limited follow-up time, which may lead to some bias in the results. Further prospective studies with multiple centers and large sample sizes are needed to guide clinical treatment |
| Other information | |  | | |
| Funding | 22 | Give the source of funding and the role of the funders for the present study and, if applicable, for the original study on which the present article is based | N/A |  |

*Give information separately for cases and controls in case-control studies and, if applicable, for exposed and unexposed groups in cohort and cross-sectional studies.

**Note:** An Explanation and Elaboration article discusses each checklist item and gives methodological background and published examples of transparent reporting. The STROBE checklist is best used in conjunction with this article (freely available on the Web sites of PLoS Medicine at http://www.plosmedicine.org/, Annals of Internal Medicine at http://www.annals.org/, and Epidemiology at http://www.epidem.com/). Information on the STROBE Initiative is available at www.strobe-statement.org.
